# Surface Acoustic Wave Nebulisation Mass Spectrometry for the Fast and Highly Sensitive Characterisation of Synthetic Dyes in Textile Samples

**DOI:** 10.1007/s13361-017-1716-x

**Published:** 2017-06-28

**Authors:** Alina Astefanei, Maarten van Bommel, Garry L. Corthals

**Affiliations:** 1 Van ’t Hoff Institute for Molecular Sciences, Faculty of Science, University of Amsterdam, Science Park 904, 1098 XH Amsterdam, Netherlands; 2 Faculty of Humanities, Conservation and Restoration of Cultural Heritage, University of Amsterdam, Johannes Vermeerplein 1, 1071 DV Amsterdam, Netherlands

**Keywords:** Ambient mass spectrometry, Surface acoustic wave nebulisation, Synthetic dyes, Textiles, Art, Conservation, Restoration

## Abstract

Surface acoustic wave nebulisation (SAWN) mass spectrometry (MS) is a method to generate gaseous ions compatible with direct MS of minute samples at femtomole sensitivity. To perform SAWN, acoustic waves are propagated through a LiNbO_3_ sampling chip, and are conducted to the liquid sample, which ultimately leads to the generation of a fine mist containing droplets of nanometre to micrometre diameter. Through fission and evaporation, the droplets undergo a phase change from liquid to gaseous analyte ions in a non-destructive manner. We have developed SAWN technology for the characterisation of organic colourants in textiles. It generates electrospray-ionisation-like ions in a non-destructive manner during ionisation, as can be observed by the unmodified chemical structure. The sample size is decreased by tenfold to 1000-fold when compared with currently used liquid chromatography–MS methods, with equal or better sensitivity. This work underscores SAWN-MS as an ideal tool for molecular analysis of art objects as it is non-destructive, is rapid, involves minimally invasive sampling and is more sensitive than current MS-based methods.

**Electronic supplementary material:**

The online version of this article (doi:10.1007/s13361-017-1716-x) contains supplementary material, which is available to authorized users.

**Graphical Abstract:**
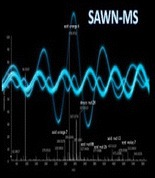
ᅟ

## Introduction

In the last 25 years, the scope and capacity of mass spectrometry (MS) for molecular analyses have been greatly expanded, its accuracy and sensitivity have been increased, and the analysis costs and time have been reduced. Currently MS relies on several methods for sample introduction, some facile, and others more technically demanding. These include electrospray ionisation (ESI), atmospheric pressure chemical ionisation, atmospheric pressure photochemical ionisation and matrix-assisted laser desorption ionisation. Their use is often specific for the type of sample that can be analysed (polarity, size range), and consequently they impose methodological challenges such as capillary clogging, and may induce in-source fragmentation and oxidation of the analytes [1]. To increase the speed of analysis and overall versatility and expand new application areas, ambient ionisation methods have been introduced in the last decade [2–4]. Their development represents a promising future for direct analysis involving simplified, faster and sensitive procedures and ultra-small amounts of sample. Additionally, little or no sample preparation or preseparation is required, which renders ambient techniques ideal for use with portable and miniaturised mass spectrometers, in addition to typical laboratory environments.

Recently, surface acoustic wave nebulisation (SAWN) was introduced as a novel ambient ionisation method [5,6]. Following SAWN, further evaporation of droplets to gaseous ions occurs in a non-destructive manner. To date we have not witnessed internal fragmentation of the molecules investigated (i.e. dyes, resins, lipids, peptides, explosives, organometallic cages). SAWN-MS has also been reported as a versatile tool for rapid analysis of biological samples [7, 8]. More recently, Tveen-Jensen et al. [9] showed that SAWN effectively interfaces with liquid chromatography (LC) for the MS analysis of a protein digests and it can be used as a lower-energy alternative to ESI for these samples. Huang et al. [7] reported SAWN is a softer method than ESI for analyte introduction from the liquid phase into the mass spectrometer. SAWN was also successfully coupled with paper-based sample delivery for the analysis of drugs in human whole blood and plasma, and of heavy metals in tap water [10]. Nevertheless, the use of SAWN as a nebulisation technique is a relatively new development and remains to be further exploited. Because of the simple setup, with SAWN there is little or no sample preparation, no need to introduce the sample into a vacuum system, capillaries or other mechanisms, or no need to involve purposely or inadvertently other chemistries. Another advantage of SAWN that we observed was that handling the liquid sample on an open substrate also minimised the contact between the liquid and its surroundings. A known problem with LC-based MS systems that contain channel walls is that fouling by debris and non-specific surface adsorption of reagents occurs.

The focus of this report is on the analysis of art objects, where invasive sampling procedures are eschewed, or at least the sampling is limited to minimally invasive events. Existing methods involving gas chromatography (GC)–MS or LC–MS require hours of sample preparation and analysis and relatively large sample amounts [11–14]. Hence, SAWN-MS was evaluated for both speed and sensitivity, both important parameters, as on-the-spot analysis would be ideal, and high sensitivity implies less invasive sampling.

Synthetic dyes are of interest in art conservation and restoration. From the nineteenth century, synthetic dyes began to replace natural dyes on the market as they were cheaper and easier to produce. Their characterisation in objects of cultural heritage, including textiles and paintings, is highly challenging because of the complexity of the materials. These include organic colourants, dye impurities and chemical instability. Additionally, a complicating factor is that during history, many different, often natural products, were used which differ in composition because of their origin, but also because of differences in preparation and application. Furthermore, organic materials are known to degrade, but knowledge of degradation products is scant. Finally, invasive sampling procedures are typically avoided when one is working with highly valued art objects, and thus only small amounts of sample can be obtained. The identification of the compounds present in different objects of art (e.g. textiles, furniture, paintings) provides valuable information regarding the intention of the artist and the meaning of the object. It can reveal information regarding the original appearance, dating and the condition of the artwork, and it can provide information about future development of the artwork or it is used in the conservation and restoration processes. In general the analysis of art objects has been limited to techniques that require relatively large sample amounts and time-consuming sample preparation procedures [12,15–19]. Several spectroscopic techniques are appealing, but they cannot provide precise identification of the compounds of interest, and also the structural information is limited. Most of the studies reported in the literature have focused on developing methods for the separation of mixtures of dyes [12,20,21], and there are very few reports [22,23] studying the composition of a dye. This is very important since each dye is a mixture of colours, and their composition and degradation affects the final colour and the discoloration or fading of art objects. Hence, there is a crucial need for analytical techniques that require exquisitely small amounts of sample, and that preserve the chemical structure of the compounds of interest during analysis, ideally avoiding degradation or inadvertent oxidation of the analytes. In our setup using SAWN, no separation columns are involved or no specific sample preparation methods are used that are compatible with separation procedures. As the sample preparation procedure occurs in the actual analysis solution, dilution or drying and reconstitution is avoided, which results in the use of extremely small amounts of sample for the MS analysis; sample preparation and analysis is in one solution. On a final note, no voltage is being applied before or during the analysis to the sample, and thus possible electrochemical oxidation of the analytes is also avoided.

## Experimental

### Chemicals

The textile samples and dye standards were provided by the Cultural Heritage Agency of the Netherlands (RCE; Amsterdam, Netherlands). Water, methanol and acetonitrile of ultra LC–MS grade were purchased from Biosolve Chimie (Dieuze, France).

### Instrumentation

All experiments were conducted with a TripleTOF 5600+ mass spectrometer (AB SCIEX, Concord, ON, Canada) and a SAWN device from Deurion (Seattle, WA, USA).

### Sample Preparation

All the analysed fibres were wool fibres dyed with synthetic dyes, and they were all subjected to the same sample treatment, as follows. Approximately 1–2 mm of fibre of the selected dyed wool samples was solubilised in 100 μL MeOH–H_2_O (1:1 v/v) and then vortexed for 2 min. A change in colour of the solution was observed after this step, indicating the extraction of the corresponding synthetic dyes.

### Surface Acoustic Wave Nebulisation Mass Spectrometry

**Figure 1:**
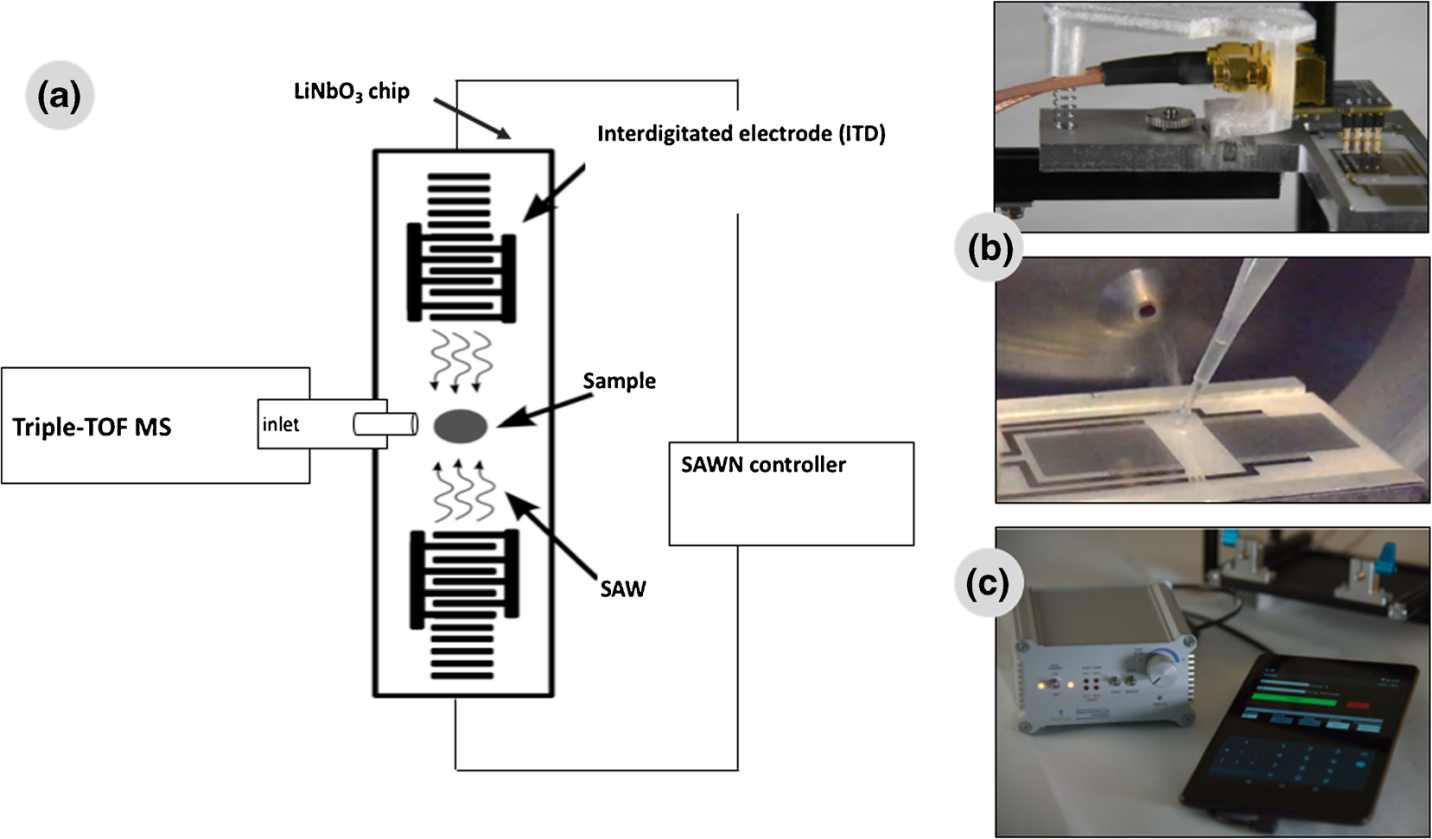
a Experimental setup, **b** chip and chip holder and **c** surface acoustic wave nebulisation (*SAWN*) controller and Android tablet with the software to run the system. *MS* mass spectrometer, *SAW* surface acoustic wave

The instrumentation and experimental setup are shown in Fig. 1. The SAWN chip was placed in front of the mass spectrometer orifice, after removal of the ESI source. The SAWN chip was placed about 1–2 cm below the mass spectrometer orifice, with the delay zone centred under the MS inlet. The liquid sample was placed directly on top of the SAWN chip near the mass spectrometer orifice (see later for the volume). With SAWN, the ionisation process starts on the chip surface (sampling stage) made of a piezoelectric material (LiNbO_3_) containing interdigitated electrodes. For MS analysis, the sampling stage is suspended opposite the mass spectrometer inlet and operated (software on Android tablet) through a connection with a frequency and power controller (see Fig. 1). For each MS analysis, SAWN was regulated by application of power to the electrodes (approximately 5 W) in continuous mode. Approximately 1 μL of sample in MeOH–H_2_O (1:1 v/v) was loaded on the chip surface, and the step was repeated up to nine times (equivalent to 10 μL loaded on the chip), and the data were accumulated in a single data file.

Data were acquired with an interface heater temperature of 150 °C, the inlet and outlet gas pressures were set at 0 psi, and the curtain gas pressure was set to the minimum valued allowed, 10 psi. The mass spectra were acquired (*m*/*z* 50–1000) in the positive/negative ionisation mode for 15–60 s with use of multichannel acquisition with an accumulation time of 3 s. The MS/MS spectra were acquired by application of a rolling collision energy (CE) to the precursor ions. PeakView 2.0 was used for data processing.

## Results and Discussion

### Surface Acoustic Wave Nebulisation

More information on the SAWN mechanism can be found elsewhere [7,8]. Briefly, application of power to the interdigitated electrodes on the chip produces a displacement within the LiNbO_3_ substrate, generating acoustic waves that propagate along the surface of the chip. This mechanical energy is transferred to the liquid droplet, overcoming its surface tension and causing it to nebulise, creating a fine mist of aerosols containing the analytes (see the video in the electronic supplementary material). Whilst undergoing further evaporation, the nebulised sample enters the vacuum interface region of the mass spectrometer, where desolvation and coulombic explosion occur, and subsequent MS analysis occurs within seconds.

### Identification of Synthetic Dyes in Textiles

It is well known that dyes are manufactured and commercialised as impure mixtures of related compounds [24]. Their identification in archaeological or historical textiles is required to investigate and understand dyeing procedures as well as for textile preservation, to reconstruct the original appearance of the artwork and to predict the stability of the colours. This is a difficult task because of the complexity of the degradation of the organic molecules, which are particularly sensitive to light [25]. At the moment, molecular understanding can be obtained only by use of invasive analytical techniques. For the past two decades, one of the most widely used methods for the analysis of natural and synthetic dyes on textiles has been high-performance LC (HPLC) with photodiode array detection (PDA) [11]. More recently, it has been improved by the combining of LC or GC methods with MS [13,14,26,27]. However, the chromatographic analysis requires the extraction of the analytes from the fibres, and milligrams of yarns have to be removed from the objects investigated [16,28]. Furthermore, derivatisation is a requisite for GC–MS analysis [26].

SAWN-MS was therefore used to detect which chromophores contribute to the final colour of dyed textiles. An ultimate goal for such analysis would be to provide information on the structure of the degradation products that might be present in the samples as a result of ageing. As a proof of concept, ten textiles dyed with a representative synthetic dye (basic or acid) were analysed. After the extraction of the dyes with a mixture of MeOH and H_2_O (1:1 v/v) (see “Experimental”), approximately 10 μL of the solution obtained was spotted on the SAWN chip, and the full MS spectra were acquired with a TripleTOF 5600+ mass spectrometer. For structural confirmation, MS/MS spectra were acquired with CE ramping (from 10 to 150 eV). Data interpretation was based on comparison of the SAWN-MS spectra of dyed textiles (provided by RCE) with the results obtained from analysis of the commercially available dye standards. Furthermore, the results were also compared with those available in the ultraperformance LC–PDA library of RCE containing approximately 250 PDA spectra of reference materials (extracted and analysed under the same conditions). Table 1 summarises negative-ion and positive-ion spectra for the ten dyes analysed by the proposed method. Expansion of this spectral library serves as a rapid analysis platform in identification and restauration.

**Table 1:** Colourants identified in the surface acoustic wave nebulisation mass spectrometry spectra of dyed wool samples

Dye, chemical formula	Assigned formula	Measured *m/z*	Theoretical *m*/*z*
Wool dyed with basic violet 3			
Basic violet 3 C_25_H_30_N_3_Cl	[M − Cl]^+^	372.2442	372.2434
Basic violet 1 C_24_H_28_N_3_Cl	[M − Cl]^+^	358.2279	358.2277
C_23_H_26_N_3_Cl	[M − Cl]^+^	344.2115	344.2121
C_22_H_24_N_3_Cl	[M − Cl]^+^	330.1956	330.1964
C_21_H_22_N_3_Cl	[M − Cl]^+^	316.1813	316.1808
C_20_H_20_N_3_Cl	[M − Cl]^+^	302.1662	302.1652
Wool dyed with basic blue 26			
Basic blue 26 C_33_H_32_N_3_Cl	[M − Cl]^+^	470.2584	470.2590
C_32_H_30_N_3_Cl	[M − Cl]^+^	456.2442	456.2434
C_31_H_28_N_3_Cl	[M − Cl]^+^	442.2269	442.2277
C_30_H_26_N_3_Cl	[M − Cl]^+^	428.2132	428.2121
Wool dyed with acid violet 7			
Acid violet 7 C_20_H_18_N_4_S_2_O_9_	[M − 2H+Na]^-^	543.0235	543.0262
	[M − H]^-^	521.0433	521.0442
	[M − 2H]^2-^	260.0179	260.0184
	[M − 2H − 2HSO_3_]^2-^	179.0533	179.0538
Ponceau 3R C_19_H_18_N_2_S_2_O_7_	[M − 2H+Na]^-^	471.0288	471.0302
Acid red 13 C_20_H_14_N_2_S_2_O_7_	[M − H]^-^	478.9598	478.9989
Acid red 26 C_18_H_16_N_2_S_2_O_7_	[M − H]^-^	457.0137	457.0145
Acid orange 7 C_16_H_12_N_2_SO_4_	[M − H]^-^	327.0436	327.0445
Direct red 28 C_32_H_24_N_6_O_6_S_2_	[M − 2H]^2-^	325.0519	325.0526
Acid red 88 C_20_H_14_N_2_SO_4_	[M − H]^-^	377.0588	377.0601
	[M − HSO_3_]^-^	297.1045	297.1033
Acid orange 6 C_12_H_10_N_2_SO_5_	[M − H]^-^	293.0243	293.0237
Acid red 18 C_20_H_14_N_2_O_10_S_3_	[M − 2H + Na]^2-^	278.9752	278.9742
Wool dyed with acid orange 6			
Acid orange 6 C_12_H_10_N_2_SO_5_	[M − H]^-^	293.0249	293.0237
Acid red 88 C_20_H_14_N_2_O_4_S	[M − H]^-^	377.0595	377.0601
	[M − HSO_3_]^-^	297.1044	297.1033
Wool dyed with acid red 88			
Acid red 88 C_20_H_14_N_2_O_4_S	[M − H]^-^	377.0596	377.0601
	[M − HSO_3_]^-^	297.1038	297.1033
Direct red 28 C_32_H_24_N_6_O_6_S_2_	[M − 2H]^2-^	325.0538	325.0526
Acid orange 6 C_12_H_10_N_2_SO_5_	[M − H]^-^	293.0247	293.0237
Acid red 18 C_20_H_14_N_2_O_10_S_3_	[M − 2H+Na]^2-^	278.9745	278.9742
Wool dyed with acid red 44			
Acid red 44 C_20_H_14_N_2_O_7_S_2_	[M − H]^-^	457.0158	457.0169
	[M − 2H + Na]^-^	479.0001	478.9989
Direct red 28 C_32_H_24_N_6_O_6_S_2_	[M − 2H]^2-^	325.0535	325.0526
Acid red 88 C_20_H_14_N_2_O_4_S	[M − HSO_3_]^-^	297.1041	297.1033
Wool dyed with acid blue 74			
Acid blue 74 C_16_H_10_N_2_O_8_S_2_	[M − H]^-^	420.9788	420.9805
	[M − 2H + Na]^-^	442.9617	442.9625
	[M − HSO_3_]^-^	341.9738	341.9742

**Figure 2:**
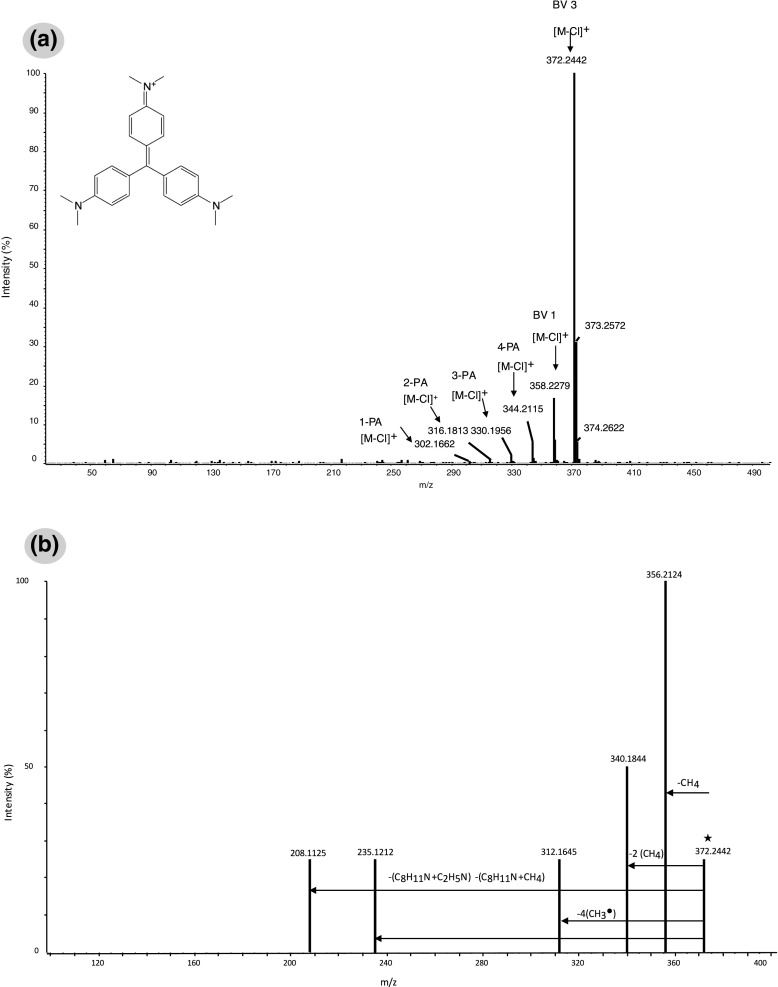
**a** SAWN mass spectrometry (MS) spectrum and **b** SAWN-MS/MS spectrum of 1-mm textile fibre dyed with basic violet 3 (*BV 3*) in MeOH–H_2_O (1:1 v/v) (collision energy 65 eV). *BV 1* basic violet 1, *1-PA* monomethyl pararosaniline, *2-PA* bimethyl pararosaniline, *3-PA* trimethyl pararosaniline, *4-PA* tetramethyl pararosaniline

Regarding the basic dyes selected for this study, triarylmethane dyes were the first ones to be produced and marketed among the early classes, and held a prominent position because of their wide variety of shades, colouring power and low costs. One of them is crystal violet, also known as basic violet 3 (BV 3) [29] or hexamethyl pararosaniline, and another important class is represented by basic blue 26, which besides the dimethylamino groups also contains a naphthylamino group. Both of them were studied in this work. The ionisation of basic dyes occurred by the loss of chloride. The SAWN-MS spectrum in positive mode of a wool fibre (approximately 1 mm) dyed with BV 3 in MeOH–H_2_O (1:1 v/v), showed the most intense peak at *m*/*z* 372.2 [M − Cl]^+^ and other peaks of lower intensity at *m*/*z* 358.2, 344.2 and 330.2 and also at *m*/*z* 316.2 and 302.2 (at trace levels) corresponding to the monodemethylated, bidemethylated, tridemethylated, tetrademethylated, pentademethylated and hexademethylated forms respectively (Fig. 2a, Table 1). The demethylated products were identified through their exact mass (less than 2 ppm accuracy). For comparison, a freshly prepared BV 3 standard solution was analysed under the same conditions. The most intense peak, [M − Cl]^+^, appeared at *m*/*z* 372.2, and the monomethylated and the dimethylated forms were observed at trace levels, probably being residues from the synthesis of this dye. As the molecule ages, the peaks corresponding to the other demethylated components appear, and their intensity increases. The more intact dye molecule is present, the more the original colour will be preserved. Furthermore, it is known that these basic dyes were often used in a mixture with various demethylated derivatives, and hence it is important to know the dye production method so as not to misinterpret the data on composition of textile samples. Nevertheless, since the ageing of these dyes results in their demethylation [30], and the presence of these compounds (even at trace levels) contributes to the discoloration and fading of works of art, the spectra obtained can be used to gain better insight into the degradation and fading of textiles dyed with these early synthetic dyes. Previous studies on BV 3 degradation were mostly performed by UV–vis spectrophotometry, HPLC–PDA and only occasionally by LC–MS [22,31]. The proposed method also allows MS/MS experiments to be performed for confirmatory purposes. As an example, Fig. 2b shows the MS/MS spectrum obtained by fragmentation of BV 3 precursor ion [M − Cl]^+^ at *m*/*z* 372.2 at a CE of 65 eV. The fragmentation pattern and the exact mass of the fragments confirm the presence of this dye as a main component in the analysed textile sample. The peaks at *m*/*z* 356.2, 342.2 and 312.2 correspond to monodemethylated, bidemethylated and tetrademethylated BV 3 ions, by the prevalent loss of CH_4_, 2CH_4_ and 4CH_3_^•^. The fragment at *m*/*z* 235.1 indicates the presence of an odd number of nitrogen atoms, as it comes from the loss of dimethylaniline and CH_4_. The smallest product ion observed, *m*/*z* 208.1, corresponds to the loss of dimethylaniline and dimethylamine. The relative abundances of and chemical structures assigned to the product ions are included in Table 2. Similarly to BV 3, the spectrum of wool dyed with victoria blue B (basic blue 26) [29] showed the most intense peak at *m*/*z* 470.2, corresponding to [M − Cl]^+^, the tetramethylated form, and also peaks corresponding to the monodemethylated, bidemethylated and tridemethylated components at *m*/*z* 456.2 442.2 and 428.2 respectively.

**Table 2:** Fragments obtained for ion precursors of basic violet 3 a.nd acid red 88

Precursor ion mass (*m*/*z*)	Product ion (*m*/*z*)	Abundance (%)
Basic violet 3, C_25_H_30_N_3_^+^ (372.2442)	C_24_H_26_N_3_^+^ (356.2124)	100
	C_23_H_22_N_3_^+^ (340.1824)	55
	C_21_H_18_N_3_^+^ (312.1645)	30
	C_16_H_15_N_2_^+^ (235.1214)	30
	C_15_H_14_N^+^ (208.1125)	30
Acid red 88, C_20_H_13_N_2_O_4_^-^ (377.0593)	C_10_H_7_NSO_3_^-^ (221.0125)	30
	C_10_H_6_SO_3_^-^ (206.0492)	5
	C_10_H_7_NO^-^ (157.0520)	<5
	C_10_H_7_O^-^ (143.0520)	8
	SO_3_^-^ (79.9600)	5

Each year, millions of kilograms of azo dyes are produced and used in diverse applications, including textile dyes, paint pigments, food colouring and printing inks. Thus, for our work several representative sulfonated azo dyes were selected for the evaluation of SAWN as an alternative rapid analysis platform (see Table 1). After the textile fibres had been immersed in the MeOH–H_2_O (1:1 v/v) mixture and vortexed for 1 min, the SAWN-MS spectra were acquired as described in “Experimental”, and were compared with the spectra obtained the commercially available synthetic dye standards for identification of compounds.

**Figure 3:**
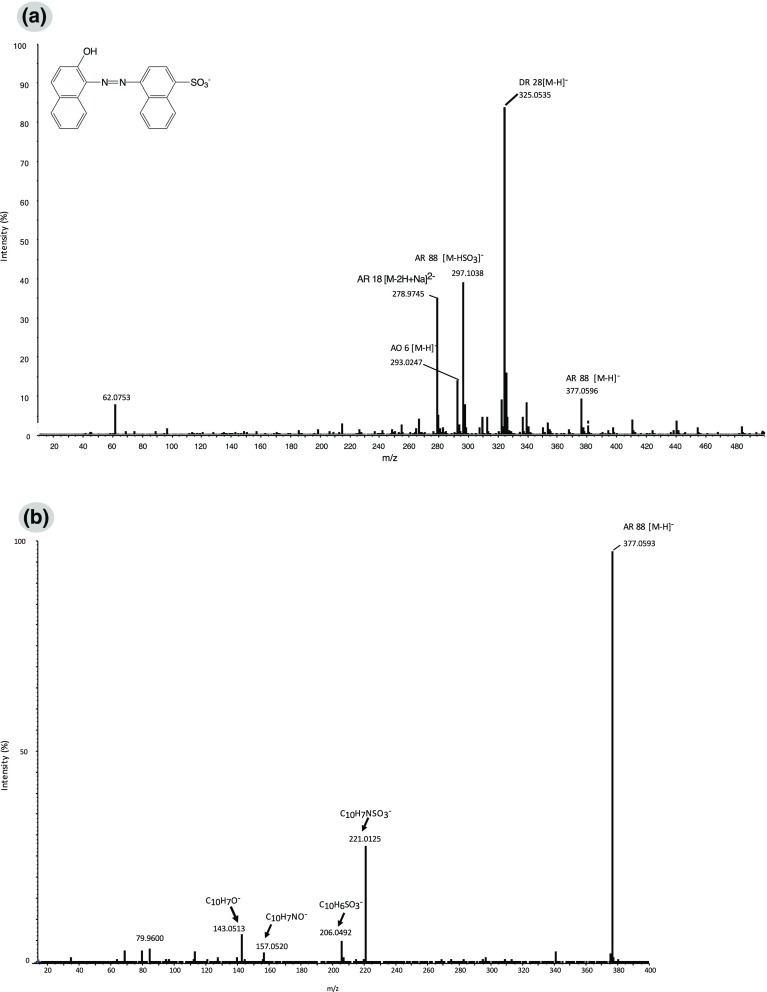
**a** SAWN-MS spectrum and **b** SAWN-MS/MS spectrum of 1-mm textile fibre dyed with acid red 88 (*AR 88*) in MeOH–H_2_O (1:1 v/v) (collision energy 150 eV). *AO 6* acid orange 6, *AR 18* acid red 18, *DR 28* direct red 28

The ionisation of the monosulfonated and bisulfonated azo dyes occurred by the loss of sodium ions and occasionally by the loss of SO_3_ group(s), giving the [M − H]^-^ and/or [M − HSO_3_]^-^ ions and also [M − 2H]^2-^ respectively (see Table 1). In general, the spectra obtained revealed the presence of a mixture of colourants. As an example, the spectrum obtained from a textile dyed with a monosulfonated azo dye, acid red 88, is shown in Fig. 3a. Besides the representative ions of this dye, [M − H]^-^ at *m*/*z* 377.0 and [M − HSO_3_]^-^ at *m*/*z*: 297.1, three other dyes (i.e. acid red 18, direct red 28 and acid orange 6) [29] were identified. The tandem MS spectrum obtained by fragmentation of the [M − H]^-^ molecular ion (*m*/*z* 377.0) with the maximum CE (150 eV) and the observed product ions are shown in Fig. 3b. The assigned chemical formulas and relative abundances are included in Table 2. As shown, even with the highest CE applied, the molecular ion’s intensity was still 100%. As expected, for this class of dyes, the cleavage of the molecule at the azo bond was observed, leading to the product ions at *m*/*z* 221.0 (C_10_H_7_NSO_3_^-^) and 157.0 (C_10_H_7_NO^-^). In addition, the cleavage before the azo group was also observed in the spectrum obtained, showing the product ions at *m*/*z* 206.0 (C_10_H_6_SO_3_^-^) and 143.0 (C_10_H_7_O^-^). The same products were observed by fragmentation of the [M − HSO_3_]^-^ molecular ion (*m*/*z* 297.1).

**Figure 4:**
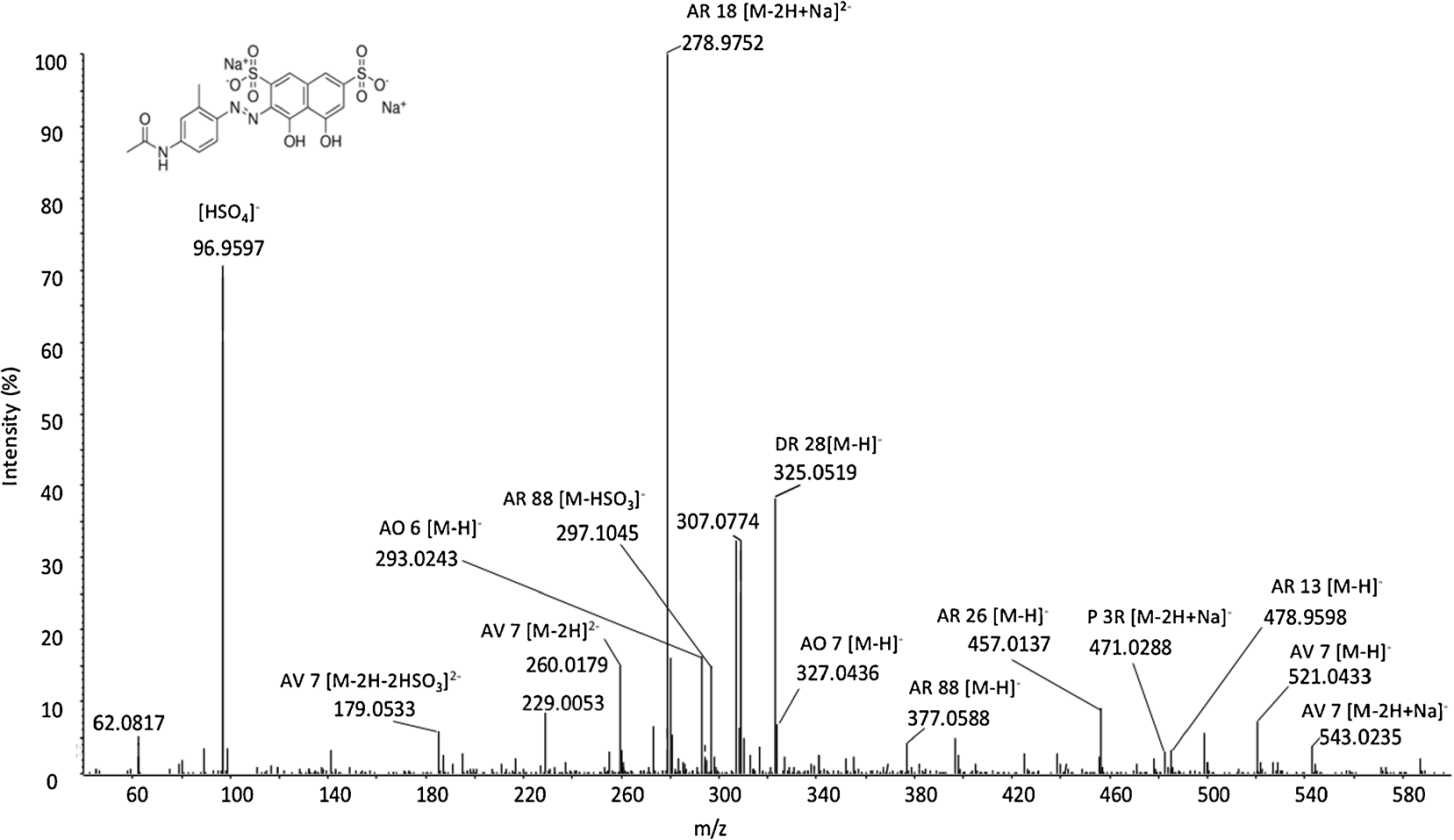
SAWN-MS spectrum of 1-mm textile fibre dyed with acid violet 7 (*AV 7*);*AO 6* acid orange 6, *AO 7* acid orange 7, *AR 13* acid red 13, *AR 18* acid red 18, *AR 26* acid red 26, *AR 88* acid red 88, *DR 28* direct red 28, *P 3R* ponceau 3R

Regarding the analysis bisulfonated azo dyestuff, the spectrum obtained for a textile fibre dyed with a representative dye, acid violet 7 [29], is depicted in Fig. 4. Besides the corresponding ion peaks at *m*/*z* 543.0, [M − 2H + Na]^-^, 521.0, [M − H]^-^, 260.0, [M − 2H]^2-^ and 179.0, [M − 2H − 2HSO_3_]^2-^, several other colourants were identified (i.e. acid red 13, 26 and 88, acid orange 7, direct red 28 and ponceau 3R) [29], and their molecular ions and *m*/*z* are included in Table 1.

For the other dyed textiles studied, the spectra obtained also revealed a mixture of at least two colours: (the corresponding molecular ions and *m*/*z* are included in Table 1). This might be because these dyes are not pure, which is often the case with early synthetic dyes, as they normally generate several side products during synthesis. To prevent carry-over, the chip was cleaned after each sample, and blanks (MeOH–H_2_O, 1:1 v/v) were acquired.

### Method Performance

The quality parameters of the method were determined, and are included in Table 3. Limits of quantitation (LOQs), based on a signal-to-noise ratio of 10:1, were obtained by analysis of successive diluted solutions of wool samples containing the compounds studied. LOQs down to 0.001 pg injected were obtained for the basic dyes and between 0.0005 and 0.5 pg injected for the acid dyes. This level of sensitivity is more than enough to detect the dye extracted from textile fibres.

**Table 3:** Quality parameters

	LOQ		Run-to run precision, RSD (%)	Accuracy, ∆_m_ (ppm)	Sample size limit (μg)
Dye, molecular ion, *m*/*z*	pg loaded	ng/L			
Basic violet 3, [M − Cl]^+^, 372.2442	0.001	1.0	6	2.1	0.5
Basic blue 26, [M − Cl]^+^, 470.2584	0.001	1.3	6	1.3	0.6
Acid violet 7, [M − 2H]^2-^, 260.0179	0.5	500	10	1.9	200
Acid orange 6, [M − H]^-^, 293.0249	0.1	100	7	4.1	150
Acid red 88, [M − H]^-^, 377.0596	0.0005	0.5	8	1.3	0.1
Acid red 44, [M − H]^-^, 457.0158	0.003	3.0	10	2.4	5.0
Acid blue 74, [M − H]^-^, 420.9788	0.2	200	9	4.1	10.0

*LOQ* limit of quantitation, *RSD* relative standard deviation

By use of the proposed method, a significant reduction in sample size compared with traditional techniques was achieved, from a few millimetres or centimetres of yarn [15,18,19] to 1–2 mm of fibre in an extremely short analysis time (15–60 s per sample). The determined sample size limits, as shown in Table 3, were between 0.1 μg (textile dyed with acid red 88) and 200 μg (textile dyed with acid violet 7). Besides the reduction in the sample size and analysis time, the extraction procedure proved to be simple, and does not require the use of additives such as HCl, trifluoroacetic acid or dimethyl sulfoxide as previously reported [18,21,22], involving the addition of a mixture of MeOH–H_2_O (1:1 v/v) to a small textile fibre and its vortexing for 1 min.

Calibration curves at concentrations between the LOQ and 1 mg/L for the corresponding dye were obtained, showing good linearity (*r* ^2^ > 0.988) for all the compounds (see Fig. S1). The run-to-run precision of the method was evaluated by analysis of five replicates of a wool sample. The relative standard deviations based on base peak intensity ranged from 6% to 10%. The accuracy, expressed as the error in mass, for the base peak of the compounds studied in the textile samples was less than 4 ppm.

## Conclusions

A new protocol for rapid, direct and efficient ambient MS using SAWN for textile samples has been developed. SAWN, as we have presented it in this report, offers unique advantages in terms of ease of implementation, non-destructive ambient conditions (the chemical structure of the analyte is preserved) and low sample amount required (i.e. high sensitivity is achieved). The results presented here exhibit for the first time the use of SAWN to separate and detect the mixture of colourants present in dyed textiles and by extension allow the investigation of the dyeing technology, and help with the understanding, restoration and conservation of textiles. The results clearly show that this technique can determine markers in dyed textiles, which can contribute to the identification, conservation and restoration of precious artworks. Furthermore, our results show significant advantages over other techniques reported in this application field, in terms of simplifying the sample extraction procedure and greatly reducing the sample size required and the analysis time (up to 1 min). The technique revealed limits of detection of femtomole levels. The chip-based format of this simple, rapid and high-sensitivity technique is already compatible for interfacing with portable mass spectrometers for field use, and is a powerful tool for facilitating high-throughput compound characterisation that could play a major role in the field of cultural heritage but also for advanced diagnostics, drug screening and environmental monitoring.

## Electronic supplementary material

Below is the link to the electronic supplementary material.

Fig. S1 Standard calibration curves for the synthetic dyes studied with *error bars. AB 74*, acid blue 74, AO 6 acid orange 6, *AR 44* acid red 44, *AR 88* acid red 88, *AV 7* acid violet 7, *BB 26* basic blue 26, *BV 3* basic violet 3 (PPTX 274 kb)

ESM 2 (MP4 6761 kb)

ESM3 (MP4 2895 kb)
