# Supplementary figures and images for: Surface Acoustic Wave Nebulisation Mass Spectrometry for the Fast and Highly Sensitive Characterisation of Synthetic Dyes in Textile Samples
Source: J Am Soc Mass Spectrom. 2017 Jun 28;28(10):2108–16. doi: 10.1007/s13361-017-1716-x (PMC5594053; doi:10.1007/s13361-017-1716-x)

## Slide 1
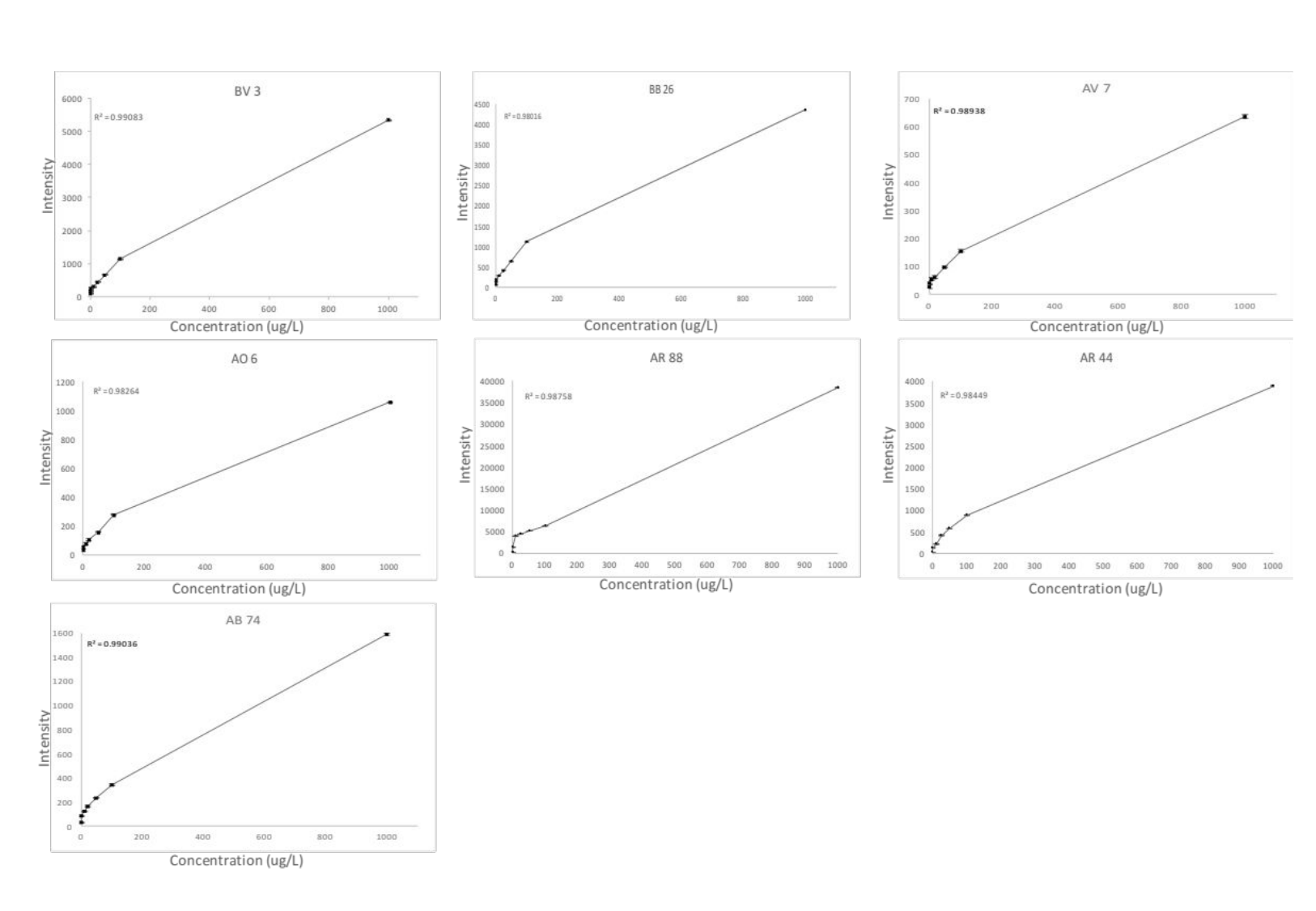

Supplement: Supplementary file 1 — Standard calibration curves for the synthetic dyes studied with error bars. AB 74, acid blue 74, AO 6 acid orange 6, AR 44 acid red 44, AR 88 acid red 88, AV 7 acid violet 7, BB 26 basic blue 26, BV 3 basic violet 3 (PPTX 274 kb) [file 13361_2017_1716_MOESM1_ESM.pptx]
